# Silica-Poly(Vinyl Alcohol) Composite Aerogel: A Promising Electrolyte for Solid-State Sodium Batteries

**DOI:** 10.3390/gels10050293

**Published:** 2024-04-25

**Authors:** João Pedro Vareda, Ana Clotilde Fonseca, Ana Cristina Faria Ribeiro, Ana Dora Rodrigues Pontinha

**Affiliations:** 1 University of Coimbra, CERES, Department of Chemical Engineering, Rua Silvio Lima, 3030-790 Coimbra, Portugal; 2 University of Coimbra, CEMMPRE, ARISE, Department of Chemical Engineering, Rua Sílvio Lima, 3030-790 Coimbra, Portugal; anafs@eq.uc.pt; 3 University of Coimbra, CQC-IMS, Department of Chemistry, Rua Larga, 3004-535 Coimbra, Portugal; anacfrib@ci.uc.pt; 4 University of Coimbra, ISISE, ARISE, Department of Civil Engineering, Rua Luís Reis Santos, 3030-788 Coimbra, Portugal; dpontinha@eq.uc.pt; 5 SeaPower, *Associação Para o Desenvolvimento da Economia do Mar*, Rua Das Acácias, Nº 40A, Parque Industrial da Figueira Da Foz, 3090-380 Figueira Da Foz, Portugal

**Keywords:** aerogel, solid electrolyte, silica, poly(vinyl alcohol), composite, solid-state batteries, sodium

## Abstract

The transition from fossil fuels is in part limited by our inability to store energy at different scales. Batteries are therefore in high demand, and we need them to store more energy, be more reliable, durable and have less social and environmental impact. Silica-poly(vinyl alcohol) (PVA) composite aerogels doped with sodium perchlorate were synthesized as novel electrolytes for potential application in solid-state sodium batteries. The aerogels, synthesized by one-pot synthesis, are light (up to 214 kg m^−3^), porous (~85%), exhibit reduced shrinkage on drying (up to 12%) and a typical silica aerogel microstructure. The formation of a silica network and the presence of PVA and sodium perchlorate in the composite were confirmed by FTIR and TGA. The XRD analysis also shows that a predominantly amorphous structure is obtained, as crystalline phases of polymer and salt are present in a very reduced amount. The effects of increasing polymer and sodium salt concentrations on the ionic conductivity, assessed via electrochemical impedance spectroscopy, were studied. At a PVA concentration of 15% (*w*/*w* silica precursors), the sodium conduction improved significantly up to (1.1 ± 0.3) × 10^−5^ S cm^−1^. Thus, this novel material has promising properties for the envisaged application.

## 1. Introduction

Electrochemical energy storage devices have gained significant attention in recent years as part of global efforts to mitigate the effects of climate change [1]. These devices play a crucial role in the transition to more sustainable and renewable energy sources, as they allow intermittently generated renewable energy to be stored. Moreover, not only has the demand for these devices increased, but their current performance cannot meet our growing needs. Employing solid-state electrolytes to produce solid-state batteries (SSBs) indeed has the potential to address the safety concerns associated with traditional liquid electrolytes and enable higher energy density and operation at high voltages [2]. 

Solid-state batteries (SSBs) are expected to revolutionize the energy storage landscape as the use of solid electrolytes offers several advantages: safety and stability under a wider range of conditions, overcoming electrolyte degradation, non-flammable, avoiding dendrite formation, and enabling metallic anodes, allowing for a substantial increase in energy density [3,4,5,6,7,8,9]. To enable better batteries, the solid-state electrolytes (SSEs) must have several properties: low cost, high ionic conductivity, wide electrochemical stability window, low electronic conductivity, low interface impedance, and be inert and environmentally friendly [6,10]. In general, there are two main categories of solid electrolytes: organic solid electrolytes and inorganic solid electrolytes. The inorganic solid electrolytes have high chemical and thermal stability, high stability with metallic anodes, are rigid, and their ionic conductivity increases with temperature [5,11,12], while organic solid electrolytes can be easily produced in larger sizes and thin films, have low ionic conductivity, are flexible, and exhibit elastic deformation [5,12,13], but still use flammable and toxic substances [14,15]. 

Polymer electrolytes have reached commercialization in lithium polymer batteries, but the drawbacks of these SSEs condition the handling and storage of the batteries [14], as explosions and the release of hazardous gases can occur [16]. Their recycling is also difficult and not very efficient, in part because lithium cannot be easily recovered even after incinerating the polymer electrolyte [14]. Recently, the research and development of solid polymer electrolytes (SPEs) has been extensively studied to further improve existing batteries [17,18,19]. However, the practical application of SSBs has been hampered by the critical interface problems between solid electrolytes and electrodes, especially inorganic solid electrolytes with high ionic conductivity [20]. 

Inorganic–polymer composite electrolytes combine the advantages of inorganic and SPEs, making them particularly suitable for the mass production of SSBs [21]. Polymer–silica composites and hybrids are gaining much attention, especially as electrolytes [22,23,24,25,26,27,28,29,30,31,32,33,34,35,36,37,38,39,40,41]. Silica provides a surface for effective anion adsorption and creates amorphous regions (e.g., decreasing polymer crystallinity) which boosts the cation’s conductivity [3,42]. Moreover, silica aerogels have gained increasing awareness due to their unique intrinsic properties, namely: ease of formation and functionalization, high specific surface area, chemical inertness, tunable pore structures, and thermal stability [43,44,45,46]. Thus, they are starting to be applied in batteries [47]. The use of silica aerogels in solid electrolytes has only very recently been reported for ions other than lithium. Incorporating the silica aerogel as a filler in the polymer matrix (as a powder or granule) has been investigated by some authors [22,23,24,25,26,27,28,29,30,31,32,33,36] to produce SPEs, mainly of poly(ethylene oxide) or poly(vinylidene fluoride). Others prepared hybrid silica aerogels, e.g., silica encapsulating ionic liquids, as electrolytes for lithium [42,48,49] and for sodium [37,50,51,52]. Silica aerogels serve as a strong backbone in a composite polymer electrolyte, improving its structural integrity, reducing polymer crystallinity, and providing a highly conductive pathway through the composite [3]. 

In this work, a composite silica–polymer aerogel doped with a sodium salt was synthesized through the one-pot sol–gel method as a novel solid electrolyte for sodium ions. Because most of the aerogel is composed of amorphous silica, which is nonflammable and has low toxicity [53], and we use nontoxic green solvents, we expect our approach to improve the chemical and thermal stabilities and safety of producing, handling and transporting the electrolyte beyond that of polymer electrolytes with silica fillers. Furthermore, silica can be degraded and recycled by processes other can combustion [53], which could improve the recovery and recycling of sodium ions from spent batteries. 

## 2. Results and Discussion

### 2.1. Properties of Aerogel Electrolytes

**Figure 1:**
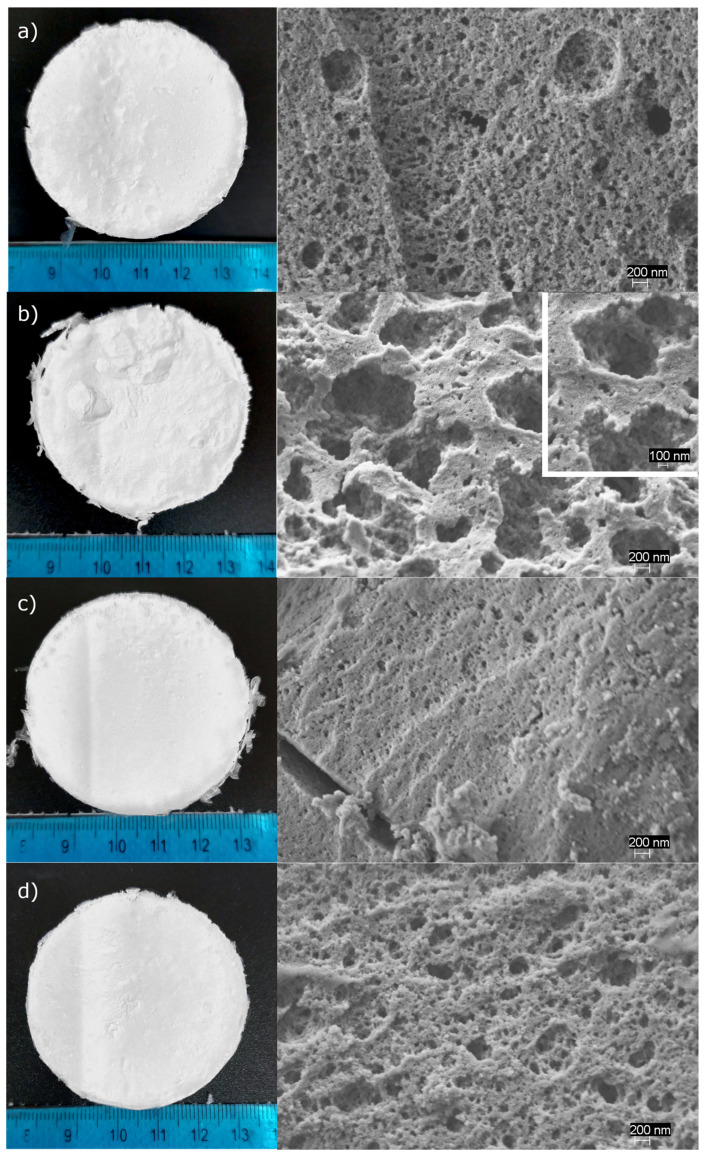
Aspect of the AEs (**left**) and SEM micrographs at 20k× magnification (**right**) for AE-10-6 (**a**), AE-10-4 and inset at 40k× magnification showing multiple pore sizes (**b**), AE-15-6 (**c**) and AE-15-4 (**d**).

Composite aerogels, hereafter referred to as aerogel electrolytes (AEs), were successfully prepared in a one-pot synthesis methodology, using silica, poly(vinyl alcohol), and sodium perchlorate. Their synthesis procedure and the meaning of the sample names are presented in Section 4. Their photographs and SEM micrographs can be seen in Figure 1. Some structural properties of the AEs, defined in Section 4.3, are listed in Table 1. 

The aerogels are white and retain their cylindrical shape and size after drying (see Figure 1). The viscosity of the sol increased with increasing concentration of the polymer and sodium salt and, as a result, air bubbles were trapped in the mixture, due to vigorous stirring, which can be seen in some SEM micrographs in Figure 1. The pearl-necklace type of structure, typical of silica aerogels, is visible in all SEM images. It can also be seen that the samples feature pores of different sizes; sample AE-15-6 (Figure 1c) has the most closed structure and individualized secondary silica particles in sample AE-10-4 (Figure 1b) are present inside the larger pores visible in the image foreground (vide inset). 

**Figure A1:**
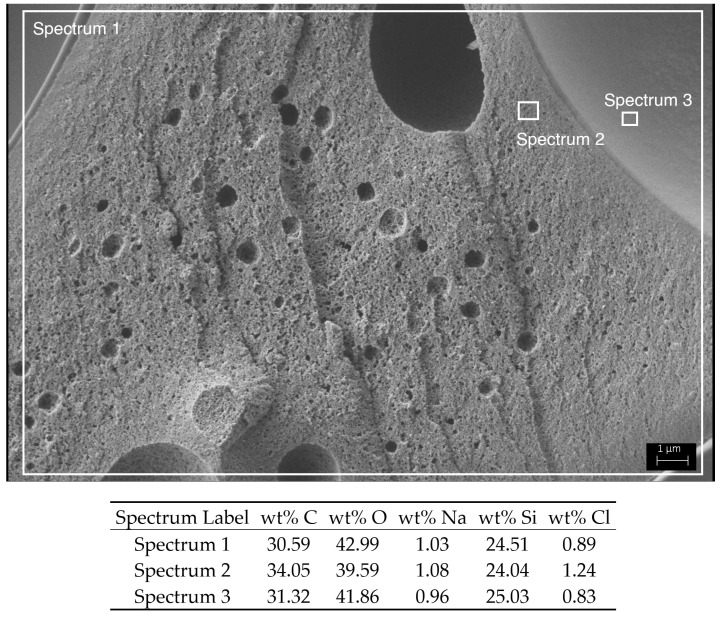
Chemical composition of AE-10-6 assessed via EDX.

**Figure A2:**
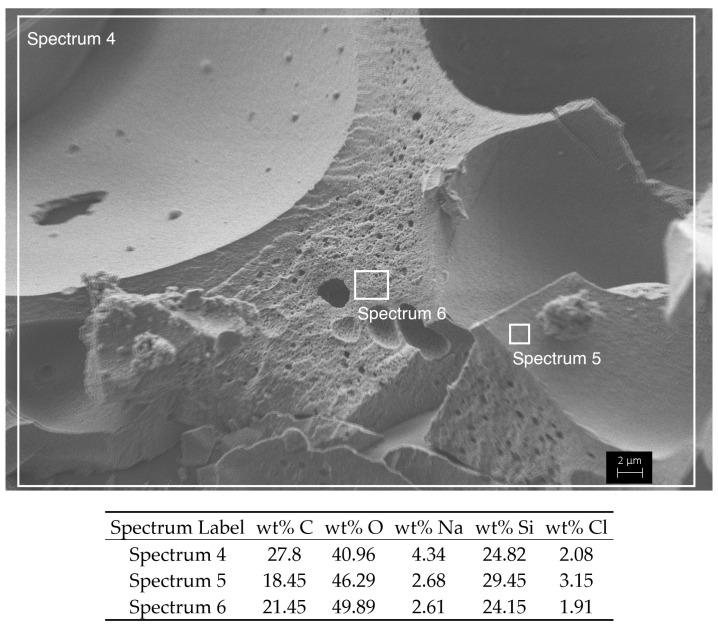
Chemical composition of AE-15-4 assessed via EDX.

The EDX results are provided in Appendix A, Figure A1 and Figure A2. Given that silicon and sodium are the most distinctive elements (C, H and O exist in the polymer and silica phases) the analysis of the results is focused on these. It is revealed that the chemical composition of the AEs is consistent across different points and very similar to that obtained over a wide area. Thus, it is not possible to distinguish between silica-rich and polymer-rich regions and thus the sample can be considered homogenous.

The analysis of Table 1 shows that all samples are very porous, with low radial shrinkage and similar porosity as well as a high specific pore volume. The AEs are very light, and their bulk density increases with increasing sodium salt concentration for the same polymer concentration and with increasing polymer concentration, as expected. The pore volume follows the same trend, with the densest sample having the lowest pore volume. All samples exhibit high BET specific surface area, which decreases with increasing polymer concentration for the tested polymer:sodium ratios. The polymer in the sol becomes entrapped in the silica gel’s pores and the former’s hydroxyl groups can form hydrogen bonds with silanol groups in the silica [46]. So, the observed result may be due to the polymer filling the pores created by the silica network and reducing the micro- and mesoporosity, which can be seen in the SEM images of Figure 1a,c. In contrast, if the amount of sodium salt is increased, the specific surface area increases in both polymer concentrations studied. It is known that alkali cations are solvated by polymeric chains [3] thus, this result can be due to the polymer having more affinity to interact with sodium ions than with silanol, leading the interpenetrating networks to arrange differently at the different polymer:salt ratios. In fact, the SEM images reveal a different microstructure for AE-10-6 and AE-15-6 than for AE-10-4 and AE-15-4. 

**Figure 2:**
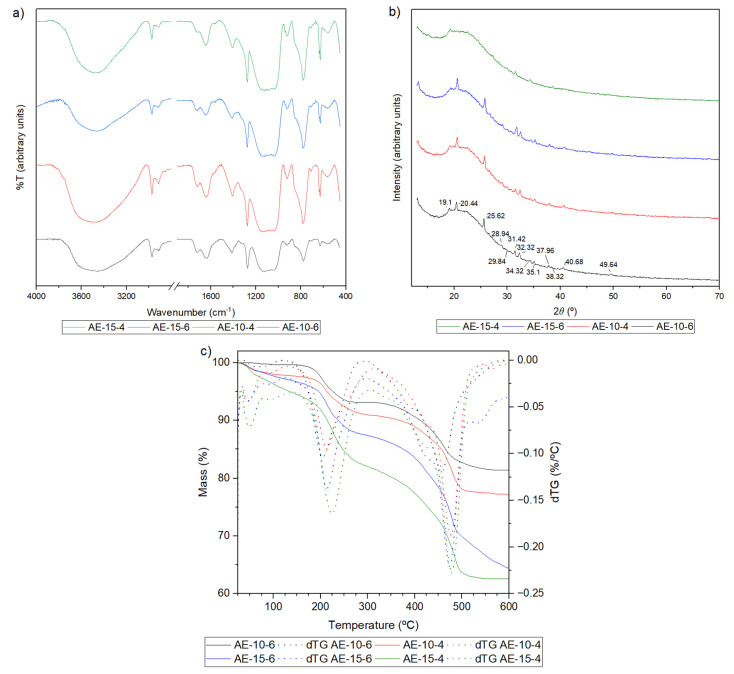
Infrared spectra (**a**), XRD diffractograms (**b**) and thermogravimetric curves (**c**) of the AEs.

The FTIR spectra of the different AEs (Figure 2a) are very similar and feature bands associated with the different reagents, revealing their incorporation in the AEs. All samples exhibit two wide and intense bands at ~3500 cm^−1^ and 1100–1000 cm^−1^. The former refers to the stretching of the hydroxyl groups in the silica and in PVA, while the latter is due to the two modes of the asymmetric stretching vibration of siloxane bonds [54]. However, in these samples, the stretching of siloxane bonds overlaps with vibrations from acetate groups in PVA (C-O-C stretching) [55] and the asymmetric stretch of perchlorate ions [56]. Bands in the 3000–2800 cm^−1^ region of the spectra are associated with the stretching vibration of C-H bonds: methyl groups in the silica matrix at ~2970 cm^−1^; acetate, methylene and methanetriyl groups in PVA at ~2920, ~2850 and ~2820 cm^−1^, respectively. The bending vibrations of these C-H bonds are visible from ~1400 to ~1280 and at ~710 cm^−1^ [54,55]. Bands associated with sodium perchlorate are visible at ~2020 and 630 cm^−1^. The carbonyl stretching vibration of acetate groups in PVA is visible at ~1720 cm^−1^. The hydroxyl groups’ bending vibration also creates a wide band at ~1640 cm^−1^. Lastly, bands at ~920 and ~780 cm^−1^ are associated with the symmetric stretching of siloxane bonds, the band at ~560 cm^−1^ is due to defects in the silica network and the shoulder at 850 cm^−1^ is due to the stretching of Si-C bond. 

**Figure A3:**
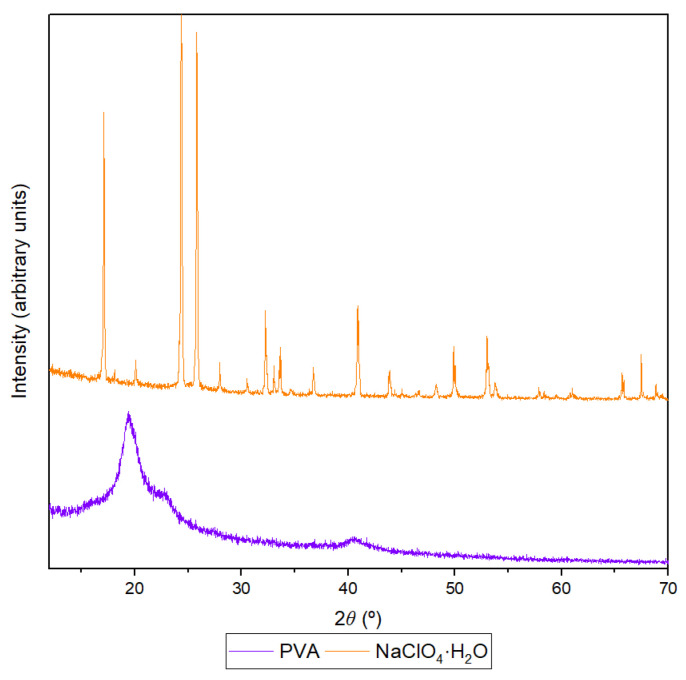
XRD diffractograms of PVA and NaClO_4_.

The XRD diffractograms are represented in Figure 2b. The samples are mostly amorphous, as expected from silica aerogels. Only when significantly increasing the signal intensity do small crystalline peaks become visible in the diffractograms, indicating that crystalline structures are present in a very small amount. It is reported in the literature that silica reduces the crystallinity of polymers [3,25] and that the sodium salt, if well dissociated, will also not exhibit crystalline phases [23,26]. In fact, the small peaks are mostly observed for 2*θ* between 20 and 40° and correlate well with those from PVA (~20, ~40°) and NaClO_4_·H_2_O (~25, ~29, ~31, ~32, ~34, ~35, ~38, ~40, ~50°). The diffractograms of these reagents are presented in Figure A3, Appendix B. The presence of these peaks indicates that a small amount of sodium perchlorate is not complexed with PVA and that the mixture between PVA and silica did not make the polymer fully amorphous. Sample AE-15-4 features virtually no peaks, suggesting a better mixture of the components in this sample, and the sample can be considered completely amorphous. The XRD results suggest a good blend between sample components, which corroborates the EDX results in which the chemical composition was consistent at different points in the sample. 

The thermogravimetric curves of the AEs are found in Figure 2c. All TG curves are similar, exhibiting three thermal degradation stages, as occurs for pure PVA [57]. The AE degradation stages occur in succession, and in some cases, the second and third stages are overlapped. As expected, the sample with the lowest amount of polymer and salt has the lowest mass loss, which increases with increasing concentration of polymer and salt. The first mass loss stage occurs from approximately 27 to 130 °C, and is associated with the evaporation of adsorbed water, residual solvents, ammonia, and the partial dehydration of sodium perchlorate monohydrate. It represents the smallest mass loss (0.4% in AE-10-6; ~2% in AE-10-4 and AE-15-6; 5% in AE-15-4). The amount of sodium salt in the AE correlates well with this mass loss. The second thermal degradation stage occurs roughly from 130 to 300 °C and is associated with the evaporation of water bound to the polymer [57] and the total dehydration of sodium perchlorate [58]. It represents ~7% of mass loss for samples AE-10-6 and AE-10-4 and 10 and 13% for samples AE-15-6 and AE-15-4, respectively. The third thermal degradation stage, occurring between 300 and 560 °C is associated with the decomposition of PVA [57] and the methyl moieties in silica [59], and the degradation of sodium perchlorate into sodium chloride [58]. Thus, it is at this stage where the highest mass loss occurs (12% in AE-10-6; 14% in AE-10-4; 23% in AE-15-6; 19% in AE-15-4). It should be noted that the mass of sample AE-15-6 is still decreasing at 600 °C, suggesting that this final degradation stage is not complete at this temperature. Also, the two final stages overlap in sample AE-15-4. 

### 2.2. Sodium Ion Conduction

**Figure 3:**
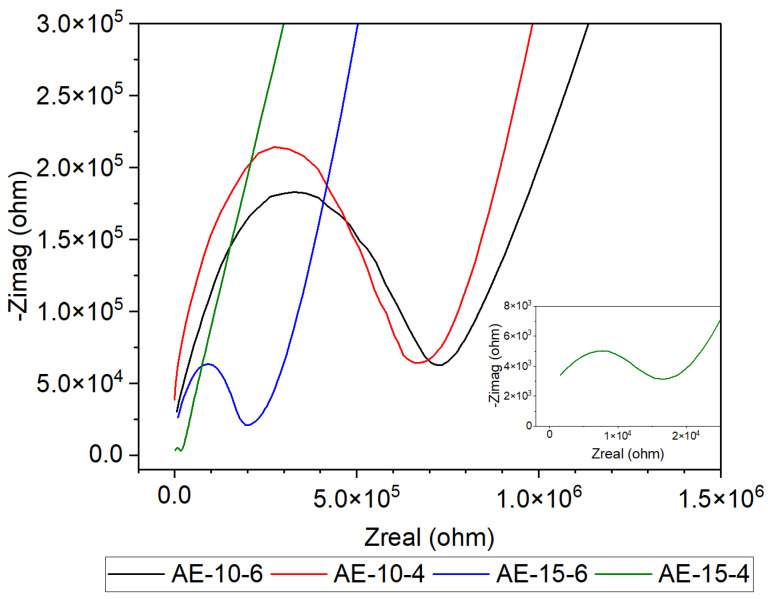
EIS spectra recorded for the different AEs. Applied potential +0.1 V and varying frequencies from 1.8 MHz to 1 Hz.

The conductivity of sodium ions through the AEs, assessed via electrochemical impedance spectroscopy, is featured in Table 2. The Nyquist curves are plotted in Figure 3 and the results of fitting these curves are in Appendix C, Table A1. We can observe that, for both polymer concentrations, the increase in salt concentration resulted in increased ionic conductivity. Thus, it seems that the polymer still had free hydroxyl groups, hence it was able to dissociate more perchlorate and interact with more sodium ions. As the polymer phase is responsible for conducting ions through the sample [3], it is no surprise that an increase in polymer concentration in the AE results in higher sodium conduction. At the 10% (*w*/*w* silica precursors) PVA concentration, the AEs had very low sodium conductivity, however, when increasing this to 15%, ionic conduction increasing one or two orders of magnitude in samples AE-15-6 and AE-15-4, respectively. It is also worth mentioning that the highest sodium ion conductivity was obtained in the sample with virtually no crystalline phases present, i.e., the sample with the highest amount of amorphous polymer. This result aligns with the belief that the ions are conducted by the amorphous regions of polymers [3]. 

Literature works [22,23,24,25,26,27,28,29] where silica aerogels have been used in solid polymer electrolytes are related only to lithium batteries and thus, a direct comparison with published literature cannot be undertaken. For the sole sake of assessing if the approach of synthesizing a polymer–silica aerogel, used in this work, has a similar performance to adding aerogel granules/powder to polymer films, the most common approach in the literature, a brief comparison is completed. The conductivity obtained in this work with sample AE-15-4 is in the same order of magnitude of some literature works [23,24,26], while it is one [25,27,29], two [22] or three [28] orders of magnitude lower in others. More similar to this work, a silica aerogel containing an ionic liquid [48], and polymer–silica aerogels, obtained by impregnation of the silica with the polymer and salt [42] or by radical polymerization of vinyl groups in the silica and in monomers [49] also showed great promise as an electrolyte for lithium batteries, with an ionic conductivity one order of magnitude higher than that of AE-15-4. We can state that the approach reported in this work is promising for the application and that further studies are required to fully prove its potential. 

The ionic conductivity at room temperature of solid sodium electrolytes containing silica nanoparticles and hybrid silica aerogel electrolytes reported in the literature is summarized in Table 3. The sodium conductivity values summarized reveal that the best result here obtained is in the same order of magnitude as those of some published works but is two orders of magnitude lower than the best ionic conductivity reported. The works in Table 3 are also based on only a few polymers. When comparing different works that use the same polymer, and incorporate silica nanoparticles, the ionic conductivity reported can differ by one order of magnitude, indicating that the silica’s type and size and the synthesis conditions play a significant role in this property. Only one work reports using PVA–silica hybrids for sodium electrolytes, with a very satisfactory result, showing that PVA can be a viable polymer to prepare a composite electrolyte. The works reporting the use of silica aerogels do not employ them in silica–polymer composites, rather the silica aerogels are used to encapsulate an ionic liquid [36,50,51] or a liquid electrolyte [52]. The authors of these studies report very high ionic conductivities. Contrarily to most authors, we chose to use aqueous based sol–gel chemistry to prepare our electrolytes because it is easy to implement, cheap, scalable and uses solvents that are not harmful to human health or the environment. Even though silica enhances the ionic conduction of polymer electrolytes, Table 3 shows that more research on the topic is required, as none of the works has the desired conductivity of 10^−2^ S cm^−1^ [3]. 

The different polymers and alkali salts used in the works cited in the previous two paragraphs could explain the different ionic conductivities reported. It should be noted that PVA is seldom reported in electrolytes but using a nontoxic, biocompatible, and biodegradable polymer can boost the safety of battery manufacturing, disassembly and recycling and promote the use of more environmentally friendly reagents. In this work, we replaced solvents like ethylene carbonate and propylene carbonate, common in the literature, with ethanol and water which have safety and environmental benefits and have used a silica backbone to improve the thermal stability of the material. 

## 3. Conclusions

We describe the synthesis of novel silica–PVA aerogel composites to be employed as a solid electrolyte in sodium ion batteries. The aerogel electrolytes retain the advantages of doping polymer electrolytes with inorganic nanoparticles reported in the literature, particularly the reduction in polymer crystallinity and the creation of pathways to improve cation conduction. However, our composite material has additional benefits: it improves nonflammability and thermal stability due to the silica matrix; is produced in a simpler way with more environmentally friendly reagents. The aerogel electrolytes feature low bulk density, high porosity, pore volume and surface area, as well as a microstructure with pores of different sizes, as is typical of silica aerogels. The formation of the silica network and the incorporation of the polymer and sodium salt are shown by FTIR spectroscopy, and XRD shows a good mixture between PVA, silica and sodium perchlorate, as the crystalline phases associated with these compounds are only present in very small amounts. At the highest polymer and sodium ion concentrations, the AE achieved a sodium conductivity comparable to works where silica aerogels or nanoparticles were used to dope polymer electrolytes, demonstrating the potential of the approach described here to produce these electrolytes. Aerogel electrolytes are still very rare in the literature, and the use of silica–polymer composite aerogels in a sodium electrolyte has not yet been reported. As such, this work contributed to expand the state of the art on solid batteries and on aerogel applications. In future work, assembling a sodium SSB using the present solid-state electrolyte and testing its recyclability and the possibility of recovering the sodium ions could be tested.

## 4. Materials and Methods

### 4.1. Materials

Tetraethyl orthosilicate (TEOS, 98%, Acros Organics, Antwerpen, Belgium) and methyltriethoxysilane (MTES, 99%, Sigma-Aldrich, Darmstadt, Germany) were used as silica sources. Anhydrous oxalic acid (p.a., ≥99%, Sigma-Aldrich) and ammonium hydroxide (25% as NH_3_, PanReac, Barcelona, Spain) were used as sol–gel catalysts. Poly(vinyl alcohol) (PVA, *M*_w_ 72,000 Da, 85–89% hydrolysis, PanReac) was selected as the polymer and sodium perchlorate monohydrate (NaClO_4_·H_2_O, 85+% as NaClO_4_, Thermo Scientific Chemicals, Waltham, MA, USA) was used as sodium salt. Ethanol (EtOH, ≥99%, Valente e Ribeiro, Belas, Portugal) and high-purity water were used as solvents. All substances were used as received. 

### 4.2. Synthesis of PVA–Silica Aerogel Electrolytes (AEs)

The silica precursors were diluted in ethanol, inside a beaker, and hydrolyzed by adding a 0.1 M oxalic acid aqueous solution, at 27 °C. After stirring the solution for 30 min, it was placed in an oven for 24 h, to complete hydrolysis. The following day, PVA and NaClO_4_ were dissolved in water, in a closed polypropylene container, at 80 °C for one hour under stirring. After complete dissolution of the solids, the mixture was allowed to cool to 50 °C and the hydrolyzed precursors, quickly followed by a 1 M aqueous solution of ammonia, were added to the polymer/salt solution, under stirring. The container was closed, and the mixture was stirred vigorously for 2 min, after which the magnetic stirrer was removed from the container and the sample was left in an oven to gel at 50 °C. The samples were allowed to age for one day at 50 °C in the oven and then dried via evaporation of the solvents at 60 °C for 3 days. 

The molar ratios of MTES:TEOS:ethanol:solvent water:acid water:basic water are 0.85:0.15:4.8:7.2:4:4. Solvent water refers to the water used as solvent for PVA and NaClO_4_, acid water refers to the water added to the synthesis with the oxalic acid solution and basic water refers to the free water added with the ammonium hydroxide solution. These ratios were kept unchanged for all samples. The amount of polymer and sodium salt was changed in accordance with the values presented in Table 4 and samples are labeled as AE-XX-YY where XX refers to the polymer mass (*w*/*w* silica precursors) and YY to the polymer:sodium molar ratio.

Two replicas of agreeing bulk density, linear shrinkage and aspect were obtained for each AE. 

### 4.3. Characterization

Bulk density (*ρ*_b_) of aerogels was obtained by weighing the samples and measuring their dimensions on the three axes. Linear shrinkage was calculated from the change in diameter of the dried sample comparatively to the gelation mold. The BET specific surface area (*S*_BET_) was obtained through nitrogen adsorption at 77 K (ASAP 2000, Micrometrics, Norcross, GA, USA). Porosity, pore volume (*V*_pore_) and average pore size (*D*_pore_) were calculated in accordance with Equations (1)–(3). The samples’ skeletal density (*ρ*_s_) was estimated to be 1300 kg m^−3^ based on the density of PVA [60] and organically modified silica aerogels [61,62,63,64].

The composite’s microstructure was observed with a field-emission scanning electron microscopy (FE-SEM) (Merlin Compact/VPCompact FESEM, Carl Zeiss Microscopy GmbH, Jena, Germany). This is equipped with an EDX spectrometer (SEM/EDX) (X-MaxN Silicon Drift EDX Detector, Oxford Instruments, Abingdon, UK) and was used to assess changes in chemical composition.

Infrared spectra of samples were obtained with KBr pellets in the wavenumber range of 4000 to 400 cm^−1^, with 128 scans and a resolution of 4 cm^−1^. The Spectrum Two FT-IR, Perkin Elmer, features a deuterated triglycine sulfate detector and a KBr beam splitter. XRD analysis was conducted in a Rigaku Smartlab, with copper radiation and Bragg–Brentano geometry, in powdered samples in a 2*θ* range of 13–70°, a step of 0.02°, a speed of 1° min^−1^, a potential of 40 kV and a current of 50 mA. TGA was carried out on a thermogravimetric analyzer (TG209 F3 Tarsus, Netzsch Instruments, Burlington, MA, USA). Samples (ca. 3.5 mg) were heated under nitrogen from 25 to 600 °C, at 10 °C min^−1^, with a flow rate of 50 mL min^−1^.

(1) $$Porosity\left(\%\right)=\left(1-\frac{\rho_{b}}{\rho_{s}}\right)\times 100$$

(2) $$V_{pore}=\frac{1}{\rho_{b}}-\frac{1}{\rho_{s}}$$

(3) $$D_{pore}=\frac{{4V}_{pore}}{S}$$

### 4.4. Electrochemical Testing

**Scheme 1:**
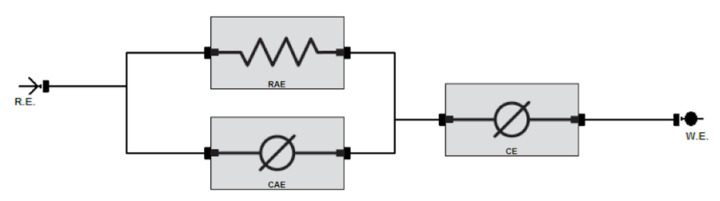
Equivalent electrical circuit used to fit the spectra.

Potentiostatic electrochemical impedance spectroscopy (EIS) was performed with a potentiostat/galvanostat/ZRA (Interface 1010E, Gamry Instruments, Warminster, PA, USA). An AC signal with an amplitude of 350 root-mean-square (rms) mV and a constant potential difference of 0.10 V, relative to the open-circuit potential, were applied to the cell. The analysis was carried in a frequency range of 1.8 MHz to 1.0 Hz, at room temperature (18 °C). The AEs were cut into flat sample pieces of regular geometry with a thickness of 2 mm and placed between two stainless steel electrodes (electrode area 1 cm^2^). Two of these sample pieces, from different parts of the aerogel, were obtained for each AE and its replica. The equivalent electrical circuit reported by Yoon, Hong and Hwang [24], represented in Scheme 1, is composed of *R*_AE_ (representing the bulk resistance of the aerogel) and *C*_AE_ (representing the capacitance of the bulk aerogel) in parallel, and in series with *C*_e_ (representing the capacitance of the electrode–aerogel interface), where *C* is constant phase elements, was used to fit the spectra. The fittings were computed with *Echem Analyst 2* (version 7.10, Gamry Instruments). The sodium conductivities (*σ*) of AEs were calculated with Equation (4), where *l* is the sample’s thickness and *A* is the contact area between the aerogel and electrode.

(4) $$\sigma =\frac{l}{R_{AE}A}$$

## Figures and Tables

**Table 1:** Physical/structural properties of the AEs.

Sample	Bulk Density (kg m^−3^)	Linear Shrinkage (%)	Porosity (%)	*S*_BET_ (m^2^ g^−1^)	*V*_pore_ (cm^3^ g^−1^)	*D*_pore_ (nm)
AE-10-6	176 ± 5	8.1 ± 0.9	86.4 ± 0.4	321 ± 10	4.9 ± 0.2	61 ± 3
AE-10-4	196 ± 8	11.5 ± 0.9	84.9 ± 0.7	339 ± 5	4.3 ± 0.2	51 ± 3
AE-15-6	194 ± 10	8 ± 2	85.1 ± 0.8	104 ± 1	4.4 ± 0.3	169 ± 10
AE-15-4	213.6 ± 0.4	8.6 ± 0.7	83.6 ± 0.0	233 ± 5	3.9 ± 0.0	67 ± 1

**Table 2:** Ionic conductivity, σ, of the AEs.

Sample	σ (S cm^−1^)
AE-10-6	(2.4 ± 0.4) × 10^−7^
AE-10-4	(3.8 ± 0.9) × 10^−7^
AE-15-6	(1.6 ± 0.3) × 10^−6^
AE-15-4	(1.1 ± 0.3) × 10^−5^

**Table 3:** Ionic conductivity at room temperature, σ, of sodium ion electrolytes containing silica.

Polymer/Ionic Liquid	Silica Form	σ (S cm^−1^)	Reference
Poly(ethylene oxide)	NP	2 × 10^−5^	[38]
Poly(ethylene glycol) grafted silica	NP	2 × 10^−5^	[39]
Poly(ethylene oxide)	NP	7 × 10^−4^	[40]
Poly (ethylene glycol)-co-ureidopyrimidinone	NP	2 × 10^−5^	[32]
Poly(ethylene glycol)	NP	9 × 10^−5^	[33]
Poly(methyl methacrylate)	NP	3 × 10^−3^	[41]
Poly(vinylidene fluoride-co-hexafluoropropylene)	NP	4 × 10^−3^	[30]
Poly(vinylidene fluoride-co-hexafluoropropylene)	NP	7 × 10^−4^	[34]
Poly(vinylidene fluoride-co-hexafluoropropylene)	NP	8 × 10^−4^	[35]
Poly(vinyl alcohol)	NP	4 × 10^−3^	[37]
Poly(ethylene oxide) + 1-butyl-3-methyl-imidazolium thiocyanate	NP	1 × 10^−4^	[31]
1-ethyl-3-methylimidazolium bis(fluorosulfonyl)imide	aerogel	5 × 10^−3^	[50]
1-Butyl-1-methylpyrrolidinium bis(trifluoromethanesulfonyl)imide	aerogel	7 × 10^−4^	[51]
Poly(vinylidene fluoride-co-hexafluoropropylene)	aerogel	9 × 10^−4^	[52]
1-ethyl-3-methylimidazolium bis(fluorosulfonyl)imide + Poly(ethylene oxide) ^1^	aerogel	2 × 10^−3^	[36]

^1^ Complex electrolyte that combines inorganic and polymer electrolytes, and an ionic liquid encapsulated in silica.

**Table 4:** Composition of the silica–PVA solid aerogel electrolytes.

Sample	Silica Mixture	Polymer Mass ^1^ (%)	Polymer:Na-Salt ^2^
AE-10-6	85% MTES 15% TEOS	10	6:1
AE-10-4		10	4:1
AE-15-6		15	6:1
AE-15-4		15	4:1

^1^ Calculated with the mass of the silica precursors. ^2^ Molar ratios calculated using the average number of vinyl alcohol groups.

## Data Availability

The datasets presented in this article are not readily available because they are under confidentiality. Requests to access the datasets should be directed to the corresponding author.

## Appendix A

The chemical composition of the AEs at different points in space, assessed via EDX, is presented in Figure A1 and Figure A2.

## Appendix B

The diffractograms of the poly(vinyl alcohol) and sodium perchlorate monohydrate used in this work, and obtained in the same way as the sample’s diffractograms, are plotted in Figure A3.

## Appendix C

The model parameters obtained upon fitting the equivalent circuit to the EIS datasets are featured in Table A1.

**Table A1:** Model parameters for the equivalent circuit.

Sample	*R*_AE_ (Ω)	*C*_AE_		*C*_e_		Goodness of Fit ^1^
		*Q* (S s^α^)	*α*	*Q* (S s^α^)	*α*	
AE-10-6	6.5 × 10^5^	3.5 × 10^−10^	0.70	8.1 × 10^−7^	0.39	5.6 × 10^−3^
AE-10-4	6.1 × 10^5^	2.0 × 10^−11^	0.86	2.5 × 10^−7^	0.48	7.5 × 10^−3^
AE-15-6	1.3 × 10^5^	2.2 × 10^−10^	0.74	1.4 × 10^−6^	0.39	3.7 × 10^−3^
AE-15-4	1.8 × 10^4^	1.4 × 10^−8^	0.54	1.6× 10^−6^	0.50	1.5 × 10^−3^

^1^ Lower value indicates better fit.
